# Radiation Response of Large-Area 4H-SiC Schottky Barrier Diodes

**DOI:** 10.3390/ma16062202

**Published:** 2023-03-09

**Authors:** Robert Bernat, Tihomir Knežević, Vladimir Radulović, Luka Snoj, Takahiro Makino, Takeshi Ohshima, Ivana Capan

**Affiliations:** 1 Ruđer Bošković Institute, Bijenička Cesta 54, 10000 Zagreb, Croatia; 2 Jožef Stefan Institute, Jamova Cesta 39, 1000 Ljubljana, Slovenia; 3 National Institutes for Quantum Science and Technology, 1233 Watanuki, Takasaki 370-1292, Japan

**Keywords:** silicon carbide, radiation detector, neutron radiation, nuclear reactor, alpha particles

## Abstract

We report on the effects of large-area 4H-SiC Schottky barrier diodes on the radiation response to ionizing particles. Two different diode areas were compared: 1 mm × 1 mm and 5 mm × 5 mm. ^6^LiF and ^10^B_4_C films, which were placed on top of the diodes, were used as thermal neutron converters. We achieved a thermal neutron efficiency of 5.02% with a ^6^LiF thermal neutron converter, which is one of the highest efficiencies reported to date. In addition, a temperature-dependent radiation response to alpha particles was presented. Neutron irradiations were performed in a JSI TRIGA dry chamber and an Am-241 wide-area alpha source was used for testing the alpha response of the 4H-SiC Schottky barrier diodes.

## 1. Introduction

Neutron detection has always been a challenge because of limited possibilities for direct detection. Possible detector media are limited to a few isotopes: He-3, Li-6, and B-10. Other isotopes with large, effective cross-sections for neutrons have several drawbacks, such as producing low-energy gamma rays or being rare isotopes that are unstable, toxic, etc. [1]. In addition, the worldwide shortage of He-3 has directed research toward Li-6 and B-10 and their use as thermal neutron converters. The detection principle is based on the conversion of neutrons to charged particles. Two common reactions are used for this conversion: ^6^Li(n, t) and ^10^B(n, α).

Silicon carbide (SiC) is a wide-band-gap semiconductor with an impressive list of properties, such as a wide band gap, a large critical electric field, high thermal conductivity, high electron saturation velocity, chemical inertness, and radiation hardness [2]. Among all SiC polytypes, 4H-SiC has received the most attention as a promising material for radiation detection in harsh environments (high temperatures and high fluences of radiation) [2,3,4]. Studies in this field have reported even better radiation resistance of 4H-SiC in high-radiation fields than silicon and germanium [5,6,7,8]. Kalinina et al. and Rao et al. have shown that 4H-SiC can work as a radiation detector up to a temperature of 300 °C [9,10].

As recently addressed in a review paper on the applications of 4H-SiC Schottky barrier diodes (SBDs) as radiation detectors, two parameters of SBDs are crucial for radiation application [11]. The first is the epitaxial layer thickness. Reported values for the epitaxial layer thickness range from a few µm to 200 µm [11,12,13,14,15,16,17]. Recently, Kleppinger et al. [14] reported on 4H-SiC radiation response using an epitaxial layer of 250 µm, which is the thickest layer ever reported. Radiation studies were performed using a 0.9 µCi Am-241 source. In our study, all 4H-SiC SBDs had an epitaxial layer thickness of 25 µm. This parameter was not been addressed within our study; we would like to refer readers interested in the influence of epitaxial layer thickness on SiC radiation response to recently published review papers and references therein [4,11]. The second parameter that is crucial for the radiation application of SiC SBDs is the area of the Schottky contact. Increasing the area of the Schottky contact increases the area sensitive to radiation. It is interesting to note that the reported values for Schottky contact areas do not differ significantly in various studies. Although it would be desirable to have very large area detectors (increased sensitivity of a single diode and, consequently, a lower limit of detection), with the increase in Schottky contact area, degradation of the electrical properties of SBDs is expected [11]. The vast majority of reported values for Schottky contacts areas range from 1 to 20 mm^2^ [11,12,13,14,15,16,17].

The efficiency of a detector is an important parameter, as it is directly correlated with the usefulness and cost-effectiveness of a detector. With the critical shortage of a rare form of helium, He-3, used for neutron detection, the finding of an (cost) efficient neutron detector is more important than ever. The efficiency of a 4H-SiC detector to thermal neutrons is limited to approximately 5% [18,19,20]. Our previously published papers have reported an efficiency of up to 4.67% for thermal neutrons using a ^6^LiF neutron converter layer [1,11].

McGregor et al. was one of the first to report the efficiency of a 4H-SiC detector using ^10^B- and ^6^LiF-coated devices, which was 4.4% [20]. Sedlačková et al. fabricated GaAs and 4H-SiC semiconductor structures capable of registering thermal neutrons. The resultant detection efficiency with a 25–30 µm thick ^6^LiF layer was 4.8% [21].

In this work, we make additional progress in the application of 4H-SiC SBDs as radiation detectors by fabricating a large-area diode (25 mm^2^). The main objective of this paper is to investigate the influence of the diode area on the radiation response. For this purpose, we compare the newly obtained results with previously published work [1,3,13] where state-of-the-art efficiency for thermal neutron detection has been reported. Moreover, we perform temperature-dependent radiation response measurements for alpha particles.

## 2. Materials and Methods

SBDs were processed on 4H-SiC n-type nitrogen-doped epitaxial layers with concentrations up to 5 × 10^14^ cm^−3^ [3]. Without a buffer layer, the epitaxial layer was grown on a silicon face toward the $\langle 11\bar{20}\rangle$ direction (8 ° off) of a 350 µm thick silicon carbide substrate (0001). While the ohmic contacts on the backside of the silicon carbide substrate were created by nickel sintering at 950 °C in an argon environment, the Schottky barrier was created by the thermal evaporation of nickel (100 nm thick) through a metal mask with patterned square holes of 1 mm × 1 mm and 5 mm × 5 mm.

**Figure 1:**
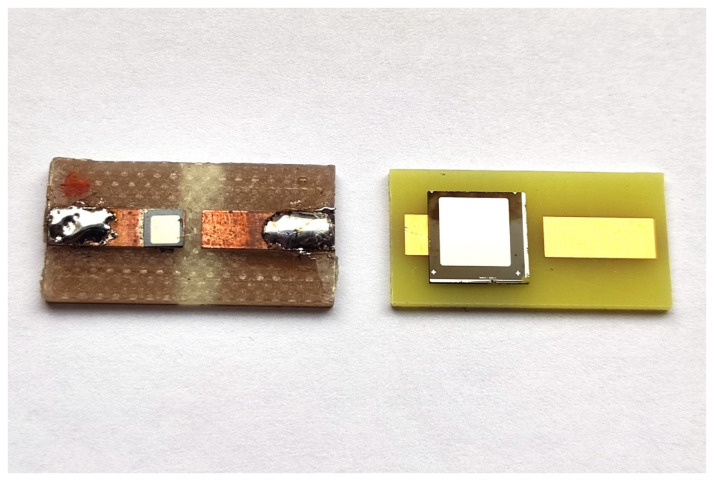
4H-SiC detectors on chip carriers with different active surface areas: (**left**) 1 mm × 1 mm and (**right**) 5 mm × 5 mm.

The SBD was mounted on a copper track with silver adhesive epoxy, while the top Schottky contact was wire-bonded to a second copper track. A coaxial cable was soldered to a printed circuit board to reverse-bias the SBD, which was also used for signal data acquisition of the radiation response. Chip carriers with 4H-SiC SBDs (Figure 1) were mounted in a 3D-printed plastic housing unit to separate the detector components under high voltage.

Current voltage (I-V) and capacitance voltage (C-V) measurements were performed using a Keithley 6487 Picoammeter/Voltage Source and a Keithley 4200 Semiconductor characterization system (Keithley Instruments, Cleveland, OH, USA) to verify the quality of the 4H-SiC SBDs prior to radiation testing.

**Figure 2:**
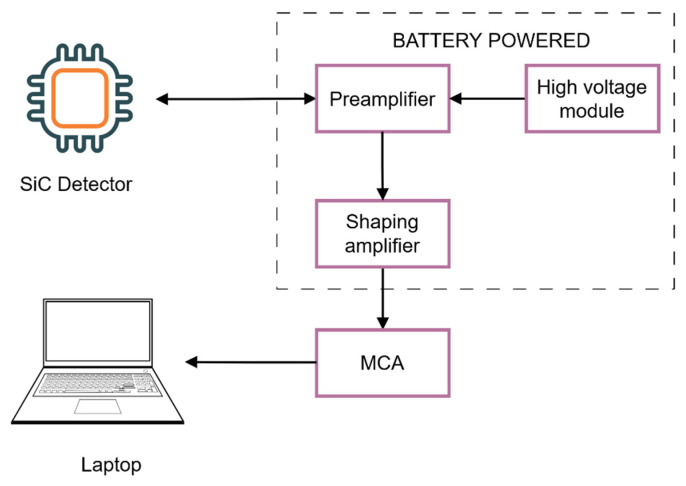
Block diagram of the detector system used in this study.

A charge-sensitive preamplifier (CREMAT CR-110), a Gaussian shaping amplifier (CREMAT CR-200-1 µs), a multichannel analyzer (AMPTEK MCA 8000D), and a laptop computer formed the detector readout system, as shown in Figure 2. The system was powered by a standalone battery power supply to minimize electronic noise in the reactor environment. The battery power supply consisted of six lithium-ion 18650 cells and two battery controllers, one for positive and the other for negative voltage. A high-voltage DC-to-DC converter (XP Power CA05P-5), which was also powered by the standalone power source, was used to provide reverse bias to the detectors. The reverse bias voltage was 100 V (compared to the back contact, a negative voltage was applied to the front contact), and the shaping time was 1 µs.

A Leybold Univex 300 (Leybold GmbH, Köln, Germany) thermal evaporation machine was used for the thin-film deposition of the converter layers. The evaporation material, ^6^LiF, was purchased as a powder, enriched in ^6^Li at 95%, and then evaporated under a vacuum onto an aluminum alloy (AlMgSi0.5) substrate. The pressure was maintained between 5 × 10^−5^ mbar and 2 × 10^−4^ mbar. The change in the oscillation frequency of a quartz crystal during the evaporation of ^6^LiF was used to track the evaporation process.

Thin films of ^10^B_4_C were deposited using a CMS-18 magnetron sputtering system (Kurt J. Lesker GmbH, Dresden, Germany) with a target that was 76.5 mm in diameter, 3.2 mm thick, and indium-bonded to a copper plate. The manufacturer of the target was RHP Technology (Seibersdorf, Austria). The mass fraction of the ^10^B isotope was 96%. The ^10^B_4_C films were deposited on an Al substrate with dimensions 20 mm × 20 mm × 0.35 mm.

For the alpha irradiations, we used a large-area Am-241 source (active area radius = 25 mm, A = 3.4 kBq). Irradiations were performed in a vacuum at different temperatures (200–390 K). 

Neutron irradiations were performed in the dry chamber of a JSI TRIGA reactor [22,23,24,25]. A graphite thermalizing column connected the dry chamber, a sizable irradiation room inside the reactor’s concrete body, with the reactor core. It was mostly used to test the radiation resistance of detectors and electronic parts. The total neutron flux φ_tot_ in the dry chamber of the reactor at 250 kW was 1.6 × 10^7^ n cm^−2^ s^−1^. The total neutron flux within the neutron energy interval of 0–5 eV φ_0–5eV_ was 8.8 × 10^6^ n cm^−2^ s^−1^.

## 3. Results

### 3.1. Electrical Characteristics of 4H-SiC Detector

**Figure 3:**
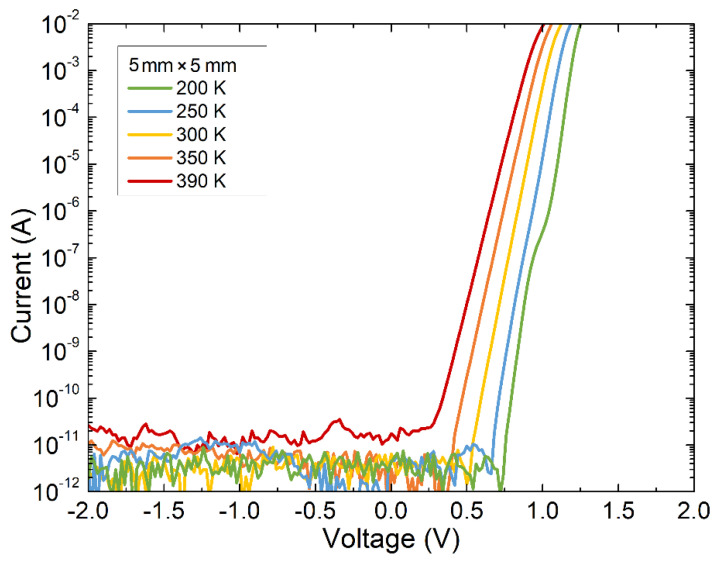
Temperature-dependent current voltage (I-V) measurement of 4H-SiC detector with active surface area of 5 mm × 5 mm. Measurements were performed in a vacuum in the temperature range from 200 K to 390 K.

The quality of the fabricated 4H-SiC SBD with an active area of 5 mm × 5 mm was assessed by current voltage (I-V) measurement (Figure 3). Measurements were carried out in a vacuum at varied temperatures from 200 K to 390 K using a cryostat. The results for the electrical characterization of a 4H-SiC SBD with an active area of 1 mm × 1 mm were published earlier [13].

From the exponential part of the current voltage characteristics, the ideality factor of 1.01 was extracted with an extremely low saturation current of ~1.4 × 10^−20^ A at the temperature of 300 K (which corresponded to a saturation current density of 5.6 × 10^−20^ A cm^−2^). The series resistance of the diode at 300 K was ~4.2 Ω (evaluated from the slope of the linear part of the I-V curve). The leakage currents for the 1 mm × 1 mm and 5 mm × 5 mm SBDs at 300K and −100 V were 9 × 10^−9^ and 2 × 10^−10^ A, respectively. At temperatures lower than 250 K, two distinct regions in the forward I-V characteristics were observed. They were attributed to Schottky barrier inhomogeneity giving rise to parallel Schottky diodes with lower barrier heights, consequently increasing the ideality factor in the low-current regime [26,27,28]. For the rest, the electric characteristics of the SBDs, such as the ideality factor and corresponding saturation current density, did not change with the increase in the active area.

**Figure 4:**
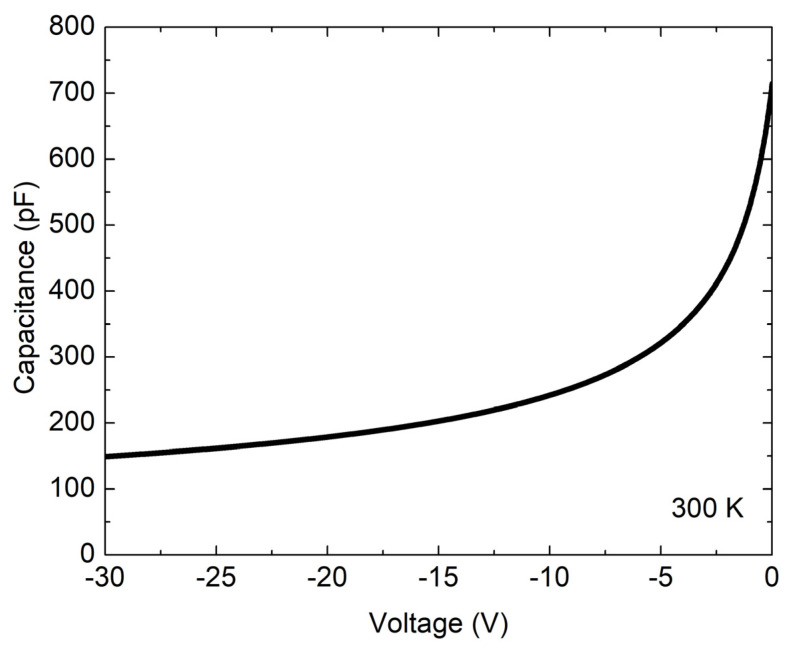
Capacitance current voltage (I-V) measurement of 4H-SiC detector with active surface area of 5 mm × 5 mm. Measurements were performed in a vacuum at 300 K.

Capacitance voltage measurements (Figure 4) were made for the 5 mm × 5 mm diode at 300 K. The measurements were performed at a frequency of 1 MHz down to a reverse bias voltage of 30 V, which was the equipment limitation. A constant doping concentration of the epitaxial layer *N_D_* of ~1.7 × 10^14^ cm^−3^ was extracted at a reverse bias of 30 V, corresponding to a depletion region width of ~14.4 µm. The depletion region width at a reverse bias voltage *V_R_* of 100 V was estimated assuming a constant doping concentration of the epitaxial layer and using the following expression [29]:

$$W_{D}=\sqrt{\frac{2\varepsilon_{SiC}\varepsilon_{0}}{qN_{D}}\left(\psi_{bi}-V_{R}-\frac{kT}{q}\right)}$$

where *ε_SiC_* is the dielectric constant for 4H-SiC, *ε*_0_ is the vacuum permittivity, *q* is the elementary charge, *ψ_bi_* is the contact potential, *T* is the temperature, and *k* is the Boltzmann constant. For a reverse bias of 100 V, the depletion region width was estimated to be 25.2 µm.

### 3.2. Radiation Response to Alpha Particles

**Figure 5:**
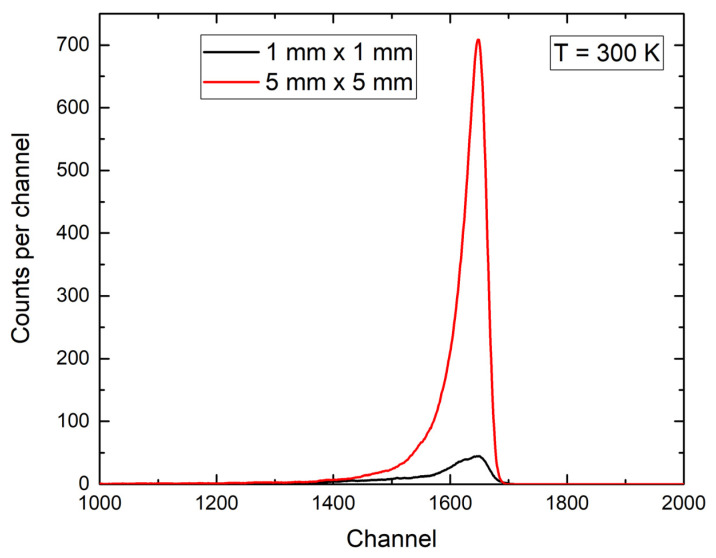
Responses of 4H-SiC detectors with different active surface areas of 1 mm × 1 mm and 5 mm × 5 mm to Am-241 alpha particles. We observed an excellent correlation of the different detector size responses to the Am-241 alpha particle energy maxima of 5486 keV (channel 1651 for 1 mm × 1 mm and channel 1648 for 5 mm × 5 mm SBDs).

The radiation response to alpha particles was measured to establish a calibration of the detector in terms of energy. We used a large-area Am-241 source with a characteristic alpha particle maxima of 5486 keV. Irradiation was performed in a cryostat under a vacuum (<0.1 mbar). The source detector distance was 3 mm. Figure 5 shows the responses of both detectors (1 mm × 1 mm and 5 mm × 5 mm) to the Am-241 source at 300 K.

The acquired spectra by the two 4H-SiC detectors showed a characteristic Am-241 alpha decay peak at 5486 keV. The energy resolution for Am-241 was 2.5%. The energy resolution was calculated from the full width at half maximum (FWHM) value. The lowest measured energy resolution for Am-241 alpha particles detected by 4H-SiC was, reportedly, as low as 0.3% [30]. 

**Figure 6:**
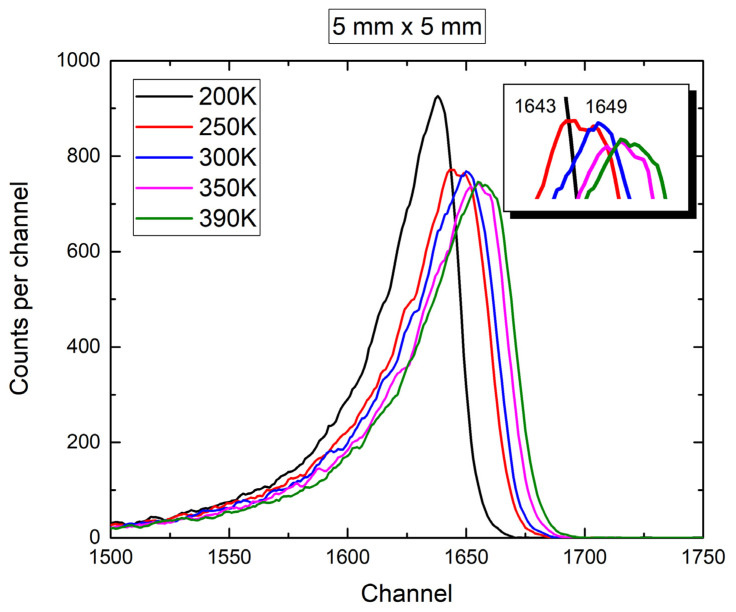
Response of 4H-SiC detector with active surface area of 5 mm × 5 mm to Am-241 alpha particles in a vacuum at different temperatures.

For the larger diode (5 mm × 5 mm) we performed measurements at different temperatures, as shown in Figure 6. The peak maximum continuously shifted on the x-scale as the radiation temperature increased (Figure 6). For all the temperature measurements except that at 200 K, we could distinguish two peaks, which corresponded to Am-241 emission energies of 5486 keV (yield 85%) and 5442 keV (yield 13%) (inset, Figure 6). The reason for only one visible peak at 200 K could not be clearly addressed, and we did not find any relevant literature data yet to support these findings. The FWHM did not change with increase in temperature. Garcia et al. reported that increase in the FHWM occurred at temperatures higher than 300 °C [31].

Temperature-dependent measurements (Figure 6) of the 4H-SiC responses to alpha particles were performed to test the quality of the 4H-SiC SBDs at different temperatures.

### 3.3. Radiation Response to Thermal Neutrons

The sensitivity of the 4H-SiC prototype detectors to thermal neutrons was tested with a JSI TRIGA reactor. For testing the radiation hardness of the detectors and electronic components, the dry chamber (an irradiation chamber inside the concrete reactor body) was ideal, as it featured relatively low but still sufficient neutron flux and, most of all, had plenty of space and was easily accessible. The detectors and preamplifiers were placed in the dry chamber, while the signal and power cables were extended to reach the reactor platform, where the remaining components were placed. At 250 kW, the dry chamber of the JSI TRIGA reactor had a total neutron flux of 1.6 × 10^7^ n cm^−2^ s^−1^.

The neutron flux for the ^6^Li(n, α)^3^H and ^10^B(n, α)^7^Li processes was 8.8 × 10^6^ n cm^−2^ s^−1^ for the neutron energy range of 0–5 eV.

**Figure 7:**
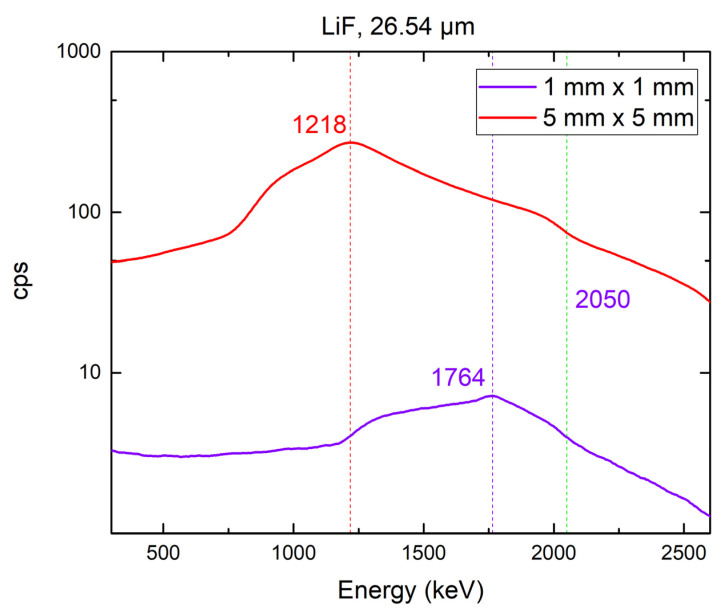
Comparison of the responses of 4H-SiC SBD detectors with different active areas (1 mm × 1 mm and 5 mm × 5 mm) equipped with a 26.54 µm thick ^6^LiF thermal neutron converter layer to the neutron field of a JSI TRIGA reactor at 250 kW.

**Figure 8:**
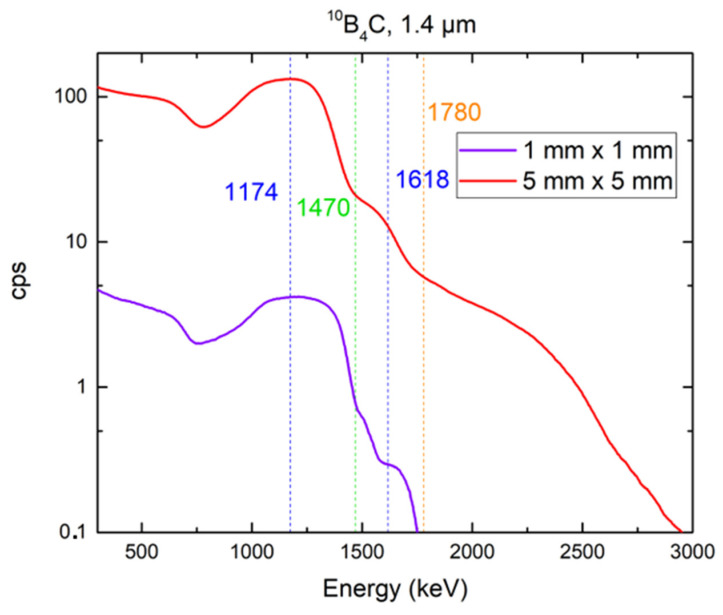
Comparison of the responses of 4H-SiC SBD detectors with different active areas (1 mm × 1 mm and 5 mm × 5 mm) equipped with a 1.40 µm thick ^10^B_4_C thermal neutron converter layer with different thicknesses to the neutron field of a JSI TRIGA reactor at 250 kW.

We used two 4H-SiC detectors (1 mm × 1 mm and 5 mm × 5 mm) equipped with thermal neutron converters of ^6^LiF (26.54 µm) and ^10^B_4_C (1.4 µm). These neutron converter thicknesses were chosen based on our previous measurements demonstrating maximum efficiency for the selected converters [13]. The responses of the 4H-SiC SBD detectors with different active areas (1 mm × 1 mm and 5 mm × 5 mm) equipped with ^6^LiF and ^10^B_4_C thermal neutron converters are shown in Figure 7 and Figure 8, respectively. An analysis of the data shown in Figure 7 and Figure 8 is covered in Section 4.

## 4. Discussion

The main aim of measuring the radiation response to Am-241 of 4H-SiC SBD detectors with two different active areas (1 mm × 1 mm and 5 mm × 5 mm) was to establish a calibration of the detectors in terms of energy. A thorough analysis of the detector responses was published earlier [13]. A vast amount of the research in this field has been conducted with smaller diodes [6,17,18,21,32]. We used the largest 4H-SiC SBD detector fabricated in this group and compared its response with that of a smaller diode. Similar to the previously published results [13], excellent energy resolution was obtained. The energy resolution for Am-241 was 2.5%.

The neutron responses of two selected detectors were measured with a JSI TRIGA reactor at 250 kW using two neutron converters: a ^10^B_4_C thermal neutron converter layer that was 1.40 µm thick and a ^6^LiF thermal neutron converter layer that was 26.54 µm thick (as shown in Figure 7 and Figure 8, respectively). We selected these converters because they showed the highest thermal neutron detection efficiencies when combined with 4H-SiC SBD detectors with smaller active areas [1]. Our previous study [1] showed that this efficiency was the highest for 3 mm × 3 mm a 4H-SiC SBD, and it was estimated as 4.67%. The 5 mm × 5 mm 4H-SiC SBD showed an even higher efficiency, which peaked at 5.02% with the use of a ^6^LiF thermal neutron converter layer (26.54 μm). The neutron response was measured at the highest reactor power (250 kW) for both the detectors used. With the increase in SBD active area, our detector system was capable of registering neutrons with a reactor power as low as 1 kW.

For the response of the detector equipped with the ^6^LiF converter, we expected a maxima attributed to the tritium produced in the ^6^Li(n, α)^3^H reaction. There was no maxima attributed to alpha particles from the above reaction since the particles were completely absorbed by the film. The shapes of the spectra for both detectors (1 mm × 1 mm and 5 mm × 5 mm) were the same, but the maxima attributed to the tritium detected by the larger diode was shifted to a lower energy (expected at 2050 keV, according to Monte Carlo simulations [1,33]). The energy at which the intensities of these maxima were lowest corresponded to the end of the motion of the detected charged particle, and most ionizations occurred at this point. For the response of the detector equipped with a ^10^B_4_C converter, we expected two maxima attributed to the tritium formed in the ^6^Li(n, α)^3^H reaction: one for the reaction in an excited state and the other for the reaction in the ground state. One maximum and one shoulder (they were too closely spaced and could not be clearly distinguished) are shown in Figure 8. A more thorough analysis of the resulting spectra was discussed in our previous paper [1]. The results presented here are in correlation with the above-mentioned previous results, which covered smaller 4H-SiC SBDs (1 mm × 1 mm, 2 mm × 2 mm, and 3 mm × 3 mm).

The maximum reported efficiency for detectors with neutron converter layers peaked at 4.4% for the ^6^LiF converter film and 4% for the ^10^B converter film [18,19,20]. We achieved a detector efficiency (based on the total counts above 500 keV [18,34]) of 1.86% for the 4H-SiC SBD equipped with a 1.40 µm thick ^10^B_4_C thermal neutron converter layer and 5.02% for the 4H-SiC SBD equipped with a 26.54 µm thick ^6^LiF thermal neutron converter layer. The efficiency of the smaller 4H-SiC SBD was negligibly smaller (less than 5% of the efficiency of the larger 4H-SiC SBD).

## 5. Conclusions

In this paper, we reported on efficient thermal neutron detection using a 4H-SiC SBD with an active area of 5 mm × 5 mm and a thermal neutron converter made of ^6^LiF with a thickness of 26.54 µm. The electric characteristics of 5 mm × 5 mm 4H-SiC SBDs, such as ideality factor and corresponding saturation current density, did not changed with the increase in the active area.

Although this was a relatively small increase in surface area compared with our previously fabricated diodes (from 9 mm^2^ to 25 mm^2^), the properties of the SBD detectors, such as efficiency, for both alpha particles and thermal neutrons remained almost the same, which gave us the certainty that we could continue to increase the active area of the SBD detectors. The results presented here showed that the active area of an 4H-SiC SBD can be increased without loss of its Schottky diode properties, as well as its detecting properties for alpha particles and thermal neutrons.

## Data Availability

Data are contained within the article.

## References

[1] Bernat R., Bakrač L., Radulović V., Snoj L., Makino T., Ohshima T., Pastuović Ž., Capan I. (2021). 4H-SiC Schottky Barrier Diodes for Efficient Thermal Neutron Detection. Materials.

[2] Kimoto T., Cooper J.A. (2014). Fundamentals of Silicon Carbide Technology.

[3] Radulović V., Yamazaki Y., Pastuović Ž., Sarbutt A., Ambrožič K., Bernat R., Ereš Z., Coutinho J., Ohshima T., Capan I. (2020). Silicon carbide neutron detector testing at the JSI TRIGA reactor for enhanced border and port security. Nucl. Instrum. Methods Phys. Res. Sect. A Accel. Spectrometers Detect. Assoc. Equip..

[4] Coutinho J., Torres V.J., Capan I., Brodar T., Ereš Z., Bernat R., Radulović V., Ambrožič K., Snoj L., Pastuović Z. (2020). Silicon carbide diodes for neutron detection. Nucl. Inst. Methods Phys. Res. A.

[5] Wright N., Horsfall A.B. (2007). SiC sensors: A review. J. Phys. D Appl. Phys..

[6] Ruddy F., Dulloo A., Seidel J., Seshadri S., Rowland L. (1998). Development of a silicon carbide radiation detector. IEEE Trans. Nucl. Sci..

[7] Seshadri S., Dulloo A., Ruddy F., Seidel J., Rowland L. (1999). Demonstration of an SiC neutron detector for high-radiation environments. IEEE Trans. Electron Devices.

[8] Ruddy F.H. (2013). Silicon Carbide Radiation Detectors: Progress, Limitations and Future Directions. Int. J. Microw. Wirel. Technol..

[9] Kalinina E.V., Ivanov A.M., Strokan N.B. (2008). Performance of p-n 4H-SiC film nuclear radiation detectors for operation at elevated temperatures (375 °C). Tech. Phys. Lett..

[10] Rao S., Pangallo G., Della Corte F.G. (2016). 4H-SiC p-i-n diode as Highly Linear Temperature Sensor. IEEE Trans. Electron Devices.

[11] Capan I. (2022). 4H-SiC Schottky Barrier Diodes as Radiation Detectors: A Review. Electronics.

[12] Ruddy F., Seidel J., Chen H., Dulloo A., Ryu S.-H. (2006). High-resolution alpha-particle spectrometry using 4H silicon carbide semiconductor detectors. IEEE Trans. Nucl. Sci..

[13] Bernat R., Capan I., Bakrač L., Brodar T., Makino T., Ohshima T., Pastuović Z., Sarbutt A. (2021). Response of 4H-SiC Detectors to Ionizing Particles. Crystals.

[14] Kleppinger J.W., Chaudhuri S.K., Karadavut O., Mandal K.C. (2021). Defect characterization and charge transport measurements in high-resolution Ni/n-4H-SiC Schottky barrier radiation detectors fabricated on 250 μm epitaxial layers. J. Appl. Phys..

[15] Flammang R.W., Seidel J.G., Ruddy F.H. (2007). Fast neutron detection with silicon carbide semiconductor radiation detectors. Nucl. Instrum. Methods Phys. Res. Sect. A Accel. Spectrometers Detect. Assoc. Equip..

[16] Lees J.E., Bassford D.J., Fraser G.W., Horsfall A.B., Vassilevski K.V., Wright N.G., Owens A. (2007). Semi-transparent SiC Schottky diodes for X-ray spectroscopy. Nucl. Instrum. Methods Phys. Res. Sect. A Accel. Spectrometers Detect. Assoc. Equip..

[17] Zaťko B., Hrubčín L., Šagátová A., Osvald J., Boháček P., Kováčová E., Halahovets Y., Rozov S.V., Sandukovskij V. (2020). Study of Schottky barrier detectors based on a high quality 4H-SiC epitaxial layer with different thickness. Appl. Surf. Sci..

[18] Manfredotti C., Giudice A.L., Fasolo F., Vittone E., Paolini C., Fizzotti F., Zanini A., Wagner G., Lanzieri C. (2005). SiC detectors for neutron monitoring. Nucl. Instrum. Methods Phys. Res. Sect. A Accel. Spectrometers Detect. Assoc. Equip..

[19] Kim H.S., Ha J.H., Park S.-H., Lee S.W., Moon M.K., Sun G.-M., Lee C.H. (2011). Characteristics of Fabricated Neutron Detectors Based on a SiC Semiconductor. J. Nucl. Sci. Technol..

[20] McGregor D.S., Hammig M.D., Yang Y.H., Gersch H.K., Klann R.T. (2003). Design considerations for thin film coated semiconductor thermal neutron detectors—I: Basics regarding alpha particle emitting neutron reactive films. Nucl. Instrum. Methods Phys. Res. Sect. A Accel. Spectrometers Detect. Assoc. Equip..

[21] Sedlačková K., Zat’Ko B., Šagátová A., Nečas V., Boháček P., Sekáčová M. (2018). Comparison of semi-insulating GaAs and 4H-SiC-based semiconductor detectors covered by LiF film for thermal neutron detection. Appl. Surf. Sci..

[22] Mandic I., Cindro V., Gorisek A., Kramberger G., Mikuz M. (2007). Online Integrating Radiation Monitoring System for the ATLAS Detector at the Large Hadron Collider. IEEE Trans. Nucl. Sci..

[23] Mandic I., Cindro V., Kramberger G., Kristof E., Mikuz M., Vrtacnik D., Ullan M., Anghinolfi F. (2004). Bulk damage in DMILL npn bipolar transistors caused by thermal neutrons versus protons and fast neutrons. IEEE Trans. Nucl. Sci..

[24] Ambrožič K., Žerovnik G., Snoj L. (2017). Computational analysis of the dose rates at JSI TRIGA reactor irradiation facilities. Appl. Radiat. Isot..

[25] Snoj L., Žerovnik G., Trkov A. (2012). Computational analysis of irradiation facilities at the JSI TRIGA reactor. Appl. Radiat. Isot..

[26] Mourya S.K., Malik G., Alisha, Kumar B., Chandra R. (2022). The role of non-homogeneous barrier on the electrical performance of 15R–SiC Schottky diodes grown by in-situ RF sputtering. Mater. Sci. Semicond. Process..

[27] Gora V., Auret F., Danga H., Tunhuma S., Nyamhere C., Igumbor E., Chawanda A. (2019). Barrier height inhomogeneities on Pd/n-4H-SiC Schottky diodes in a wide temperature range. Mater. Sci. Eng. B Solid-State Mater. Adv. Technol..

[28] Im H.-J., Ding Y., Pelz J.P., Choyke W.J. (2001). Nanometer-scale test of the Tung model of Schottky-barrier height inhomogeneity. Phys. Rev. B Condens. Matter Mater. Phys..

[29] Sze S.M., Ng K.K. (2006). Physics of Semiconductor Devices.

[30] Chaudhuri S., Krishna R., Zavalla K., Mandal K. (2013). Schottky barrier detectors on 4H-SiC n-type epitaxial layer for alpha particles. Nucl. Instrum. Methods Phys. Res. Sect. A Accel. Spectrometers Detect. Assoc. Equip..

[31] Garcia T.R., Kumar A., Reinke B., Blue T.E., Windl W. (2013). Electron-hole pair generation in SiC high-temperature alpha particle detectors. Appl. Phys. Lett..

[32] Ruddy F.H., Dulloo A.R., Seidel J.G., Palmour J.W., Singh R. (2003). The charged particle response of silicon carbide semiconductor radiation detectors. Nucl. Instrum. Methods Phys. Res. Sect. A Accel. Spectrometers Detect. Assoc. Equip..

[33] Ziegler J.F., Ziegler M.D., Biersack J.P. (2010). SRIM–The stopping and range of ions in matter (2010). Nucl. Instrum. Methods Phys. Res. Sect. B Beam Interact. Mater. Atom.

[34] Giudice A.L., Fasolo F., Durisi E., Manfredotti C., Vittone E., Fizzotti F., Zanini A., Rosi G. (2007). Performances of 4H-SiC Schottky diodes as neutron detectors. Nucl. Instrum. Methods Phys. Res. Sect. A Accel. Spectrometers Detect. Assoc. Equip..

